# Orthogonal tuning of gene expression noise using CRISPR–Cas

**DOI:** 10.1093/nar/gkaa451

**Published:** 2020-06-01

**Authors:** Fan Wu, Jiyoung Shim, Ting Gong, Cheemeng Tan

**Affiliations:** Department of Biomedical Engineering, University of California Davis, Davis, CA 95616, USA; Department of Biomedical Engineering, University of California Davis, Davis, CA 95616, USA; Department of Biomedical Engineering, University of California Davis, Davis, CA 95616, USA; Department of Biomedical Engineering, University of California Davis, Davis, CA 95616, USA

## Abstract

The control of gene expression noise is important for improving drug treatment and the performance of synthetic biological systems. Previous work has tuned gene expression noise by changing the rate of transcription initiation, mRNA degradation, and mRNA translation. However, these methods are invasive: they require changes to the target genetic components. Here, we create an orthogonal system based on CRISPR-dCas9 to tune gene expression noise. Specifically, we modulate the gene expression noise of a reporter gene in *Escherichia coli* by incorporating CRISPR activation and repression (CRISPRar) simultaneously in a single cell. The CRISPRar uses a single dCas9 that recognizes two different single guide RNAs (sgRNA). We build a library of sgRNA variants with different expression activation and repression strengths. We find that expression noise and mean of a reporter gene can be tuned independently by CRISPRar. Our results suggest that the expression noise is tuned by the competition between two sgRNAs that modulate the binding of RNA polymerase to promoters. The CRISPRar may change how we tune expression noise at the genomic level. Our work has broad impacts on the study of gene functions, phenotypical heterogeneity, and genetic circuit control.

## INTRODUCTION

Gene expression is noisy due to environmental fluctuations and the stochasticity of biochemical reactions that involve a small number of molecules. The gene expression noise impacts all biological systems (1–3). It causes cell-to-cell variations in temporal and spatial manners, leading to a difference in cell states, phenotypes, and fates of an isogenic population (2–4). The gene expression noise can be either beneficial or deleterious. For example, the stochasticity in gene expression can affect cell fate determination in both prokaryotic (5) and eukaryotic cells (4,6,7). For prokaryotic cells, gene expression noise can enhance the fitness of a population under fluctuating or stressful environments (8–10). In addition, the noise may introduce uncertainties and disorders in a regulated biological process and lead to certain diseases (11,12). Furthermore, gene expression noise is generally undesirable for synthetic genetic circuits because it diminishes the precise control of the circuits (13). Therefore, the tuning of gene expression noise so that it can be harnessed in some cases but reduced in other cases, remains an important question in synthetic biology and the study of cellular noise (14).

Previous studies have tuned gene expression noise by modulating transcription initiation, mRNA degradation, and translation rate (15–17). For example, Murphy *et al.* have tuned expression noise, while decoupling it from expression mean, in yeast by engineering GAL1 promoter and its operators (15,16). Schmiedel *et al.* have used microRNA to inhibit translation rate and increase mRNA degradation to reduce noise for a lowly expressed gene (17). Recent work has controlled transcription and mRNA degradation rates using inducers to tune noise (18). Besides modulating the biochemical reaction rates, researchers have also found that cell-to-cell variations are affected by the architecture of gene regulatory networks (19,20). For instance, a previous study has shown that negative autoregulation can reduce the heterogeneity of gene expression (21). The previous studies have indeed revealed design rules and principles for the modulation of expression noise. However, these strategies require the manipulation of endogenous genetic components, such as promoters or regulatory networks. Can we tune gene expression noise without changing the endogenous genetic components? Such an orthogonal approach for tuning noise may enable the flexible modulation of any gene expression of interest.

Here, we develop a CRISPR activation and repression (CRISPRar) tool that modulates the gene expression noise of a reporter gene in *E. coli*. The CRISPRar tool uses an identical functionalized dCas9 that recognizes two types of single guide RNAs to achieve gene activation and repression simultaneously in the same bacterium. We design a library of sgRNA variants that exhibit various activation and repression strengths. Combining pairs of sgRNA in the same bacterium, we find that the CRISPRar tool can tune the gene expression noise or mean independently. Based on our results, we generate empirical rules underlying the CRISPRar tool. Our work presents a novel and orthogonal tool that can tune the gene expression noise and mean. The work may have broad impacts on the study of phenotypical heterogeneity, cell fate determination, and dynamic control of synthetic genetic circuits.

## MATERIALS AND METHODS

### Bacterial strains and chemicals (M1)

**Figure 1.**
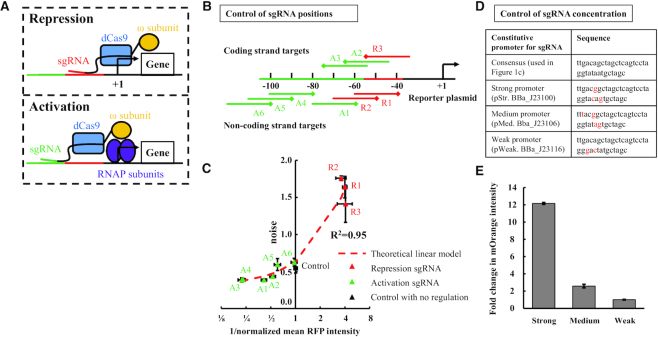
Gene activation and repression using a functionalized dCas9 protein. (**A**) dCas9ω can work as both expression activator and repressor. When the dCas9ω is close to the transcriptional start site (+1), it can repress the transcription of a gene (top). When the dCas9ω is at an appropriate distance from the transcriptional start site, the ω subunit can stabilize the assembly of RNAP and enhance the transcription. (**B**) To characterize the effect of sgRNA targeting positions on the transcriptional regulation of dCas9ω, we create a library of nine sgRNA variants (R1–R3 and A1–A6. See Supplementary Table S1 for the sequences). The sgRNAs guide the dCas9ω protein to either the coding strand or the non-coding strand of the reporter plasmid. Diamond shapes represent the PAM positions of the sgRNA variants. (**C**) The noise (linear scale) versus 1/normalized mean expression (log_2_ scale) shows a strong linear correlation (*R*^2^ = 0.95), which agrees with an existing linear model (red line). The sgRNA variants of R1–R3 repress gene expression (colored as red thereafter). In contrast, the sgRNA variants of A1–A6 activate gene expression (colored as green thereafter). Each error bar represents the standard error of the mean (SEM) with *n* = 6. (**D**) List of constitutive promoters that control the expression of sgRNA. Red letters indicate mutated nucleotides from the consensus promoter. (**E**) The strength of each constitutive promoter is measured using mOrange expression. The fold change is calculated by normalizing mOrange intensity with that of the weak promoter. The error bars are SEM with *n* = 6.

All experiments were performed using a rpoZ knock-out *Escherichia coli* MG1655 strain, which was a generous gift from Dr Jesse Zalatan's lab at University of Washington. For the characterization of constitutive promoter strength (Figure 1D and E), we cloned a mOrange gene under the designed constitutive promoters from the Anderson promoter collection (BBa_J23100, BBa_J23106 and BBa_J23116. See Figure 1D for sequences) in a pET15b vector. For the CRISPRa and CRISPRr constructs, we used three compatible plasmid vectors in the *E. coli* strain. The dCas9ω was expressed from a medium copy number plasmid (pBR322 ORI) with an arabinose-induced promoter. The sgRNA was transcribed from a medium copy number plasmid (p15A ORI) with the constitutive promoter. When two sgRNAs were transcribed in the same direction for CRISPRar constructs, a 300 nt length spacing including two transcription terminators was placed in between. The reporter RFP gene was cloned in a low copy number plasmid (pSC101 ORI) under a weak constitutive promoter (BBa_J23117). The synthetic PAM targeting region (the colored region in Figure 1B. See Supplementary Table S1 for the sequence) was adapted from previous work (22) and cloned upstream to the promoter of the RFP gene. All engineered strains were maintained as glycerol stocks at –80°C for long-term storage or on LB agar plates at 4°C for short-term storage.

Before each experiment, *E. coli* strains were grown overnight at 37°C in Luria Broth (LB) (VWR) with appropriate antibiotics (kanamycin sulfate at 30 μg/ml, carbenicillin at 100 μg/ml and chloramphenicol at 200 μg/ml working concentration). The fresh overnight cultures were diluted 1:1000 into the M9 minimal medium (VWR) supplemented with 0.2% glucose and 0.2% casamino acids with appropriate antibiotic selection and grown at 37°C on a shaker for 2 h. For the CRISPRa and CRISPRr constructs, arabinose was also supplemented at 0.025% working concentration. Carbenicillin and chloramphenicol were purchased from Sigma. Kanamycin sulfate was purchased from Amresco. Arabinose was purchased from Sigma.

### Measurement of mOrange using a platereader (M2)

Bacteria were pre-grown in M9 medium as described. 200 μl of bacterial culture was aliquoted into a black flat-bottom 96-well microplate (Corning Costar). Time series of OD_600_ and mOrange were measured using Tecan M1000Pro platereader at 37°C with shaking (orbital, 20 s every min) for 14 h. The excitation and emission for the mOrange were 548 and 562 nm, respectively.

### Measurement of expression mean and noise of RFP using flow cytometry (M3)

Bacteria containing CRISPRa and CRISPRr constructs were pre-grown in M9 medium as described. 200 μl of bacterial culture was aliquoted into a 96-well plate. The bacterial cultures were incubated in the platereader at 37°C with the same shaking protocol as described before. After 14 h, bacterial cultures were diluted into 4% PFA with 1:200 dilution and stored on ice before flow cytometry. Flow cytometry was performed on Thermo Fisher Attune NxT flow cytometer equipped with an excitation filter at 561 nm and emission filter at 620/15 nm. Pure 4% PFA was included as a blank to gate-out unspecific signal based on FSC and SSC. At least 20,000 events were collected. The mean (μ) and standard deviation (σ) of RFP intensity in the samples were calculated from the measurements. The expression noise (*η*^2^) was then calculated as ${{{\eta }}^2} = \frac{{{{{\sigma }}^2}}}{{{{{\mu }}^2}}}$ . Bacteria that only contained dCas9ω and RFP reporter without any sgRNA were included as a control in each experiment for data normalization.

### Statistical test (M4)

All statistical tests were performed using six replicates. To compare the statistical means of two groups, a one-tail *t*-test was used with *P* < 0.05, because we compared the groups in a specific direction (e.g. from lower to higher values). For a group of three or more, the statistical means were compared using a one-way ANOVA test. When comparing noise from two categories (Figures 3B and C), we identified data points that exhibited similar average expression intensity (±0.03 difference in 1/normalized mean RFP intensity) from each category and performed a two-way ANOVA test.

### Theoretical model and parameter estimation (M5)

To understand the tuning of gene expression noise using CRISPRar, we used a simple model that assumed the promoter of a gene could exhibit either ON- (*D*_on_) or OFF-state (*D*_off_). The transitions between *D*_on_ and *D*_off_ were governed by two reaction rate constants: k_on_ and k_off_. The analytical solutions of expression mean and noise already existed for the model (23). Specifically, the expression mean of a protein could be approximated using ${{{\rm mean}}}\ = \ {{\rm C}} \times \frac{{{{{\rm k}}_{{{{\rm on}}}}}}}{{{{{\rm k}}_{{{{\rm on}}}}} + {{{\rm k}}_{{{{\rm off}}}}}}}$, where *C* was a constant in our model that depended on translation rate, mRNA, and protein degradation rate. The expression noise could be approximated as ${{{\eta }}^2} = \frac{1}{{{{{\rm mean}}}}}\ + \frac{{{{{\rm k}}_{{{{\rm off}}}}}}}{{{{{\rm k}}_{{{{\rm on}}}}}}} \cdot \frac{1}{{{{\tau }} \cdot ( {{{{\rm k}}_{{{{\rm on}}}}} + {{{\rm k}}_{{{{\rm off}}}}}} ) + 1}},$ where τ was the degradation rate constant of the protein. When we compared the noise of two groups (${{\bf \eta }}_1^2\ {{\rm vs}}.{{\bf \eta }}_2^2$), one assumption we made was the mean values were the same, which led to $\frac{{{{{\rm k}}_{{{\rm on}},1}}}}{{{{{\rm k}}_{{{\rm on}},1}} + {{{\rm k}}_{{{\rm off}},1}}}} = \frac{{{{{\rm k}}_{{{\rm on}},2}}}}{{{{{\rm k}}_{{{\rm on}},2}} + {{{\rm k}}_{{{\rm off}},2}}}}\ \rightarrow \frac{{{{{\rm k}}_{{{\rm off}},1}}}}{{{{{\rm k}}_{{{\rm on}},1}}}} = \frac{{{{{\rm k}}_{{{\rm off}},2}}}}{{{{{\rm k}}_{{{\rm on}},2}}}}$. As a result, we only needed to consider the term $\frac{1}{{{{\bf \tau }} \cdot ( {{{{\rm k}}_{{{\rm on}}}} + {{{\rm k}}_{{{\rm off}}}}} ) + 1}}$ when comparing ${{\bf \eta }}_1^2\ {{\rm vs}}.{{\bf \eta }}_2^2$ if we assumed the means were the same. In summary, the model allowed us to approximate relative level of expression mean and noise based on k_on_ and k_off_.

**Figure 2.**
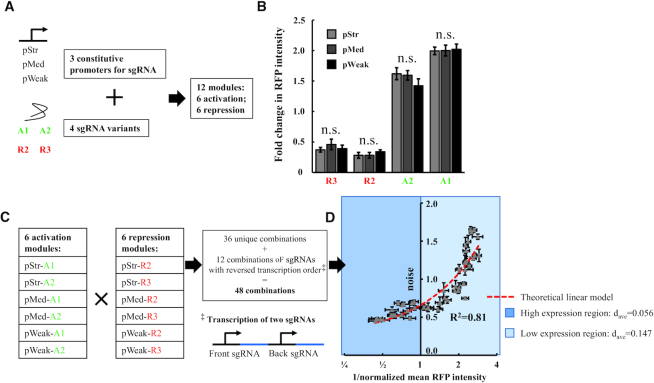
Quantification and combination of dCas9 activation and repression modules. (**A**) Three constitutive promoters and four sgRNA variants (from Figure 1) are selected to form a library of CRISPRa and CRISPRr modules. The library has a total of 12 modules: six activation (three promoters combine with A1 & A2) and six repression (three promoters combine with R2 & R3) modules. (**B**) We measure RFP expression regulated by each module using a flow cytometer. The fold change is calculated by normalizing to a positive control that contains no sgRNA. We find that the expression mean of RFP is different between A1, A2, R2 and R3. However, the expression mean of RFP is not significantly different between pStr, pMed and pWeak. The error bars are SEM with *n* = 6. The n.s. represents no significant difference from ANOVA test (*P*> 0.3 for all cases). (**C**) We combine the six activation and six repression modules to form 48 CRISPRar constructs. 36 of the constructs are unique combinations (six activation times six repression modules). Because two sgRNAs are transcribed in sequential order from a plasmid, we add 12 more constructs by switching the transcription order of the sgRNAs (See Supplementary Figure S1 for the list of all CRISPRar constructs). (**D**) We plot the expression noise (y-axis on linear scale) versus the inverse of the expression mean (x-axis on log_2_ scale) for all 48 CRISPRar constructs. The expression noise of the CRISPRar constructs is less linearly correlated with the inverse of the expression mean (*R*^2^ = 0.81). We separate the constructs into either high expression region (dark blue) or low expression region (light blue).

For simplicity, we assumed that the CRISPRr and CRISPRa only affected the k_on_ and k_off,_ respectively. Thus, we approximated ${{{\rm k}}_{{{\rm on}}}} = {{{\rm k}}_{{{\rm on}},{{\rm wt}}}}\ ( {1 - \frac{{{{{\rm k}}_{{\rm r}}}[ {{\rm R}} ]}}{{{{{\rm K}}_{{\rm r}}} + [ {{\rm R}} ]}} \cdot {{\rm in}}{{{\rm t}}_1}} )$ and ${{{\rm k}}_{{{\rm off}}}} = {{{\rm k}}_{{{\rm off}},{{\rm wt}}}}\ ( {1 - \frac{{{{{\rm k}}_{{\rm a}}}[ {{\rm A}} ]}}{{{{{\rm K}}_{{\rm a}}} + [ {{\rm A}} ]}} \cdot {{\rm in}}{{{\rm t}}_2}} )$ where k_on,wt_ and k_off,wt_ were the wild-type ON- and OFF-rate constants with no regulation (assumed both constants were 1). [A] and [R] were relative concentrations of two sgRNAs in the CRISPRar constructs. We assumed that the sgRNA pool in a single cell had a capacity of 1. The capacity was shared by two sgRNAs based on the strength of the constitutive promoter of the sgRNA. Two constants k_r_ and k_a_ were estimated using the experimental results from CRISPRa or CRISPRr module (Figure 2B). For example, if the concentration of [R] was saturated for CRISPRr only, k_on_ became k_on,wt_ (1 – k_r_). Thus, the expression fold change measured in experiment for CRISPRr could be approximated as $\frac{{{{{\rm k}}_{{{\rm on}},{{\rm wt}}}}( {1 - {{{\rm k}}_{{\rm r}}}} )}}{{{{{\rm k}}_{{{\rm on}},{{\rm wt}}}}( {1 - {{{\rm k}}_{{\rm r}}}} ) + {{{\rm k}}_{{{\rm off}},{{\rm wt}}}}}}/\frac{{{{{\rm k}}_{{{\rm on}},{{\rm wt}}}}}}{{{{{\rm k}}_{{{\rm on}},{{\rm wt}}}} + {{{\rm k}}_{{{\rm off}},{{\rm wt}}}}}}$, allowing the estimation of k_r_. A similar estimation was applied to k_a_. We assumed that when the CRISPRa and CRISPRr targeted the same DNA strands, they competed for the same binding sites. The competition was modeled using Michaelis-Menten kinetics with competitive inhibition. Specifically, ${{{\rm K}}_{{\rm r}}} = {{{\rm K}}_{{{\rm ro}}}}\ ( {1 + \frac{{[ {{\rm A}} ]}}{{{{{\rm K}}_{{\rm i}}}}}} )$, ${{{\rm K}}_{{\rm a}}} = {{{\rm K}}_{{{\rm ao}}}}\ ( {1 + \frac{{[ {{\rm R}} ]}}{{{{{\rm K}}_{{\rm i}}}}}} )$ and ${{\rm in}}{{{\rm t}}_1} = {{\rm in}}{{{\rm t}}_2}\ = \ 1$. When the CRISPRa and CRISPRr targeted different DNA strands, one might reduce the activity of another through interactions. The interactions were described using ${{\rm in}}{{{\rm t}}_1} = \frac{1}{{1 + {{{\rm K}}_{{{\rm int}}1}}[ {{\rm A}} ]}}$, ${{\rm in}}{{{\rm t}}_2} = \frac{1}{{1 + {{{\rm K}}_{{{\rm int}}2}}[ {{\rm R}} ]}}$, ${{{\rm K}}_{{\rm r}}} = {{{\rm K}}_{{{\rm ro}}}}$ and ${{{{\rm K}}}_{{{\rm a}}}} = {{{\rm K}}_{{{{\rm ao}}}}}$. The values of the parameters used in the model analysis were $\tau = {\rm{ }}1,{\rm{ }}{\rm K_{\rm ro}} = {\rm{ }}0.01,{\rm{ }}{\rm K_{\rm ao}} = {\rm{ }}0.02,{\rm{ }}{\rm K_{\rm i}} = {\rm{ }}10,{\rm{ }}{\rm K_{\rm int1}} = {\rm{ }}0.5,{\rm{ }}{\rm K_{\rm int2}} = {\rm{ }}2$. The description of the parameters is summarized in Supplementary Table S5.

## RESULTS

### Validate the basic noise vs. mean model using CRISPR-dCas9

We first design a CRISPR-dCas9 system in a rpoZ knock-out strain of *E. coli* and track the expression of a red fluorescent protein (RFP) from a low copy number plasmid. The rpoZ gene encodes ω subunit of RNA polymerase (RNAP). The lack of the ω subunit in *E. coli* does not affect the cell viability (24) but allows us to activate gene expression using a dCas9 with a C-terminal fused ω subunit (25,26). The reporter RFP is regulated by a constitutive promoter that contains a synthetic sequence region based on a previous study (22) (Figure 1A. See Supplementary Table S1 for the sequence). The synthetic region contains several PAM sites, which allow us to control the binding distance of dCas9ω–sgRNA complex to the transcriptional start site. A prior work (26) has shown that when a sgRNA is designed to guide the dCas9ω close to the promoter, the dCas9ω can block the binding of RNAP, leading to gene repression (Figure 1A, top). When the dCas9ω forms a complex with a sgRNA at an appropriate distance from the promoter, the fused ω subunit can stabilize the assembly of RNAP, leading to gene activation (24) (Figure 1A, bottom). As a result, this system allows us to either activate or repress RFP expression by tuning the binding distance of an identical dCas9ω from the promoter using different sgRNAs.

We next validate a classical model of gene expression noise using the designed CRISPR–dCas9 system. This model states that the gene expression noise (*η*^2^), defined as ${{{\eta }}^2} = \frac{{{{{\sigma }}^2}}}{{{{{\mu }}^2}}}$ where σ is the standard deviation and μ is mean of gene expression within a bacterial population, is linearly correlated with the inverse of the gene expression mean. To test the model, we implement individual sgRNAs that target DNA sequences at distinct positions from the promoter. We create a library of nine sgRNA variants that target either coding or non-coding strand of the reporter plasmid (A1–A6 and R1–R3 in Figure 1B. See Supplementary Table S1 for the sgRNA sequences). The sgRNA variants are transcribed from a constitutive promoter (consensus promoter in Figure 1D). The PAM sites of the sgRNA variants range from 40 to 100 bp upstream of the transcriptional start site (see Supplementary Table S1). The gene expression noise and mean are calculated using the RFP intensity from flow cytometry. We find that the expression mean of RFP can be modulated by varying the target-position of the sgRNAs in our system (Figure 1C). When the target-position is within ∼40–60 bp upstream from the transcriptional start site, the expression mean of RFP is repressed (R1–R3 in Figures 1B and C). In contrast, when the target-position is more than ∼60 bp upstream from the transcriptional start site, the expression mean of RFP can be increased (A1–A6 in Figure 1B and C). We find that the activation of the expression reaches the maximum level at ∼80 bp upstream from the transcriptional start site (A3–A4 in Figure 1C). The expression mean declines when the sgRNA targets the DNA sequences further from the transcriptional start site (A5–A6 in Figure 1C). This observation agrees with the general expectation that an optimal position is required for gene activation by dCas9ω (26). Furthermore, we find that the expression noise is linearly correlated to the inverse of the expression mean (*R*^2^ = 0.95 in Figure 1C). The linear correlation suggests that the promoter behaves following the behavior of a constitutive promoter with the first-order degradation of RNA and proteins (27). The findings support the feasibility of simultaneous expression activation and repression using identical functionalized dCas9 in *E. coli*. They also demonstrate the power of the CRISPR–dCas9 system in modulating gene expression noise and mean without changing the target genetic module in *E. coli*.

### Establish the control of CRISPRar tool using sgRNA target-position and concentration

Even though CRISPR–dCas9 system has been tested using single sgRNAs, this study represents the first time when repressing and activating CRISPR–dCas9 are combined to target the same promoter in a single bacterium. Thus, we investigate two control strategies before combining the activation and repression (the combinations are referred to as CRISPRar constructs thereafter). The two control strategies are target-position and concentration of sgRNA. For the control of sgRNA target-position, we select four sgRNA variants from the characterized library (A1, A2, R2 and R3 in Figure 1C) for two reasons. First, the selected sgRNAs target either the coding (A1 and R2) or non-coding (A2 and R3) strands. Second, the selected sgRNAs can form a combination of two sgRNAs with ∼0–20 bp overlaps in their target DNA sequences. We speculate that the overlapped target sequence is critical for achieving competition between CRISPR activation (CRISPRa) and CRISPR repression (CRISPRr). For the control of sgRNA concentration, we select three constitutive promoters from the Anderson promoter collection (BBa_J23100, BBa_J23106 and BBaJ23116, http://parts.igem.org) with different transcription strength (For simplicity, we use pStr, pMed, and pWeak to represent BBa_J23100, BBa_J23106, and BBa_J23116 respectively. Figure 1D). We clone the selected promoters upstream of a mOrange gene to quantify their strength. We find that the three promoters exhibit different strength: the strong promoter (pStr. BBa_J23100) exhibits around 5-fold and 12-fold higher mOrange intensity than the medium promoter (pMed. BBa_J23106) and the weak promoter (pWeak. BBa_J23116) respectively (Figure 1E). The characterization of the underlying control mechanisms allows us to implement CRISPRar in the subsequent experiments.

Based on the characterization of sgRNA-position and concentration controls, we pair the three promoters with the four selected sgRNA variants to form a combinatorial library of promoter-sgRNA modules. The library consists of 12 modules in total (six CRISPRa modules and six CRISPRr modules. Figure 2A). We next test the activity of each module. We find that the RFP expression mean of each sgRNA pair is different when the sgRNA-position varies (A1, A2, R2 and R3. Figure 2B). However, the RFP expression mean is not significantly different when the sgRNA concentration varies (pStr, pMed and pWeak. Figure 2B). The results suggest that the amount of sgRNA is likely saturated for activation or repression with all three promoters. The quantification results also allow us to estimate several key parameters of a mathematical model in subsequent experiments (Figure 5 and see Method Section M5 for details of the model).

### Reveal the empirical rules of CRISPRar for the tuning of gene expression noise

After the characterization of the CRISPRa and CRISPRr modules, we create a library of CRISPRar constructs by mixing the characterized modules. Here, we clone one activating and one repressing promoter-sgRNA modules into the same plasmid to form a total of 48 combinations (Figure 2C and Supplementary Figure S1). We find that noise is less linearly correlated to the inverse of the mean (*R*^2^ = 0.81. Figure 2D) when compared to the regulation using single sgRNAs (*R*^2^ = 0.95. Figure 1C). The deviation from the linear correlation suggests that the promoter now operates following a two-state (ON and OFF) model, as has been shown in the past for regulated promoters (28,29). The deviation of the CRISPRar constructs from the theoretical linear model also implies the decoupling of expression noise from mean. This decoupling is necessary for the tuning of noise independent from mean. To quantify the deviation, we calculate the average distance (*d*_ave_) from the experimental points of the CRISPRar constructs (grey dots in Figure 2D) to the predicted linear model (red line in Figure 2D). We find that the CRISPRar constructs at low expression region (light blue region in Figure 2D) show a larger deviation (*d*_ave_ = 0.147) than the CRISPRar constructs at high expression region (dark blue region in Figure 2D. *d*_ave_ = 0.056). This result implies the decoupling of noise from mean is more profound at the low expression region.

**Figure 3.**
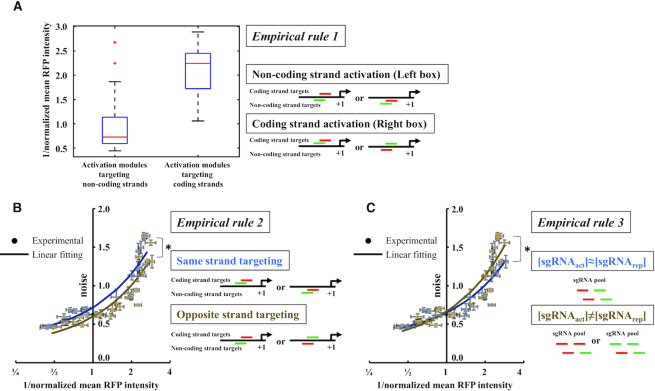
Empirical rules of CRISPRar for the tuning of gene expression noise. (**A**) Empirical rule 1: the CRISPRar constructs are separated into two groups: CRISPRa that targets non-coding strand (left box) and coding strand (right box). The boxplot shows that the constructs with non-coding-strand targeting CRISPRa generally have higher expression mean (smaller 1/normalized mean intensity) than the ones with coding-strand targeting CRISPRa. (**B**) Empirical rule 2: the CRISPRar constructs are separated into two groups: same strand targeting (blue dots) and opposite strand targeting (yellow dots). The same-strand targeting constructs (blue line) exhibit higher noise than the opposite-strand targeting constructs (yellow line). The error bars represent the SEM from *n* = 6. An asterisk indicates *P* < 0.05 from a two-way ANOVA. (**C**) Empirical rule 3: the CRISPRar constructs are separated into two groups: [sgRNA_act_] ≈ [sgRNA_rep_] (blue dots) and [sgRNA_act_] ≠ [sgRNA_rep_] (yellow dots). The noise of the [sgRNA_act_] ≈ [sgRNA_rep_] constructs (blue line) is below the [sgRNA_act_] ≠ [sgRNA_rep_] constructs (yellow line) at low expression region. The error bars represent the SEM from *n* = 6. An asterisk indicates *P* < 0.005 from a two-way ANOVA.

Next, we reveal three empirical rules for the tuning of expression noise using CRISPRar (Figure 3). For empirical rule 1, we classify the CRISPRar constructs into two groups based on whether the CRISPRa modules target non-coding DNA strand or coding DNA strand (Schematic in Figure 3A). We find that when the CRISPRa targets the non-coding DNA strand, the expression mean is generally higher than the CRISPRa that targets coding DNA strand (Boxplot in Figure 3A). This empirical rule may be used to control the expression mean of the CRISPRar constructs.

For empirical rule 2, we group the CRISPRar constructs that have two modules targeting either the same DNA strand or the opposite DNA strand (Schematic in Figure 3B). Linear fitting of the experimental data is used to visualize the relationship between two groups. We find that the constructs that target the same DNA strand generally exhibit higher expression noise than the ones that target the opposite DNA strand (the blue line is above the dark yellow line at all expression regions in Figure 3B). The result suggests that the competition of CRISPRa and CRISPRr at the same strand leads to increased expression noise (two-way ANOVA with *P*-value < 0.05).

For empirical rule 3, we investigate the impact of sgRNA concentrations on the tuning of expression noise. The two sgRNAs in CRISPRar constructs are transcribed from two different promoters. We assume that the concentrations of two sgRNAs are roughly equal when the promoters have the same strength (combinations of two pStr, two pMed, or two pWeak. Schematic in Figure 3C). In contrast, when the promoters have different strengths, we assume that the concentration of two sgRNAs is not equal (Schematic in Figure 3C). At a low expression region, the noise of the unequal [sgRNA] group (dark yellow line in Figure 3C) is above the equal [sgRNA] group (blue line in Figure 3C). At a high expression region, the noise of the unequal [sgRNA] group is approximately the same as the equal [sgRNA] group. We conclude that the tuning of expression noise based on the sgRNA concentrations is more profound at the lower expression region (two-way ANOVA with *P*-value < 0.005).

### The expression mean and noise of RFP can be tuned independently using CRISPRar

**Figure 4.**
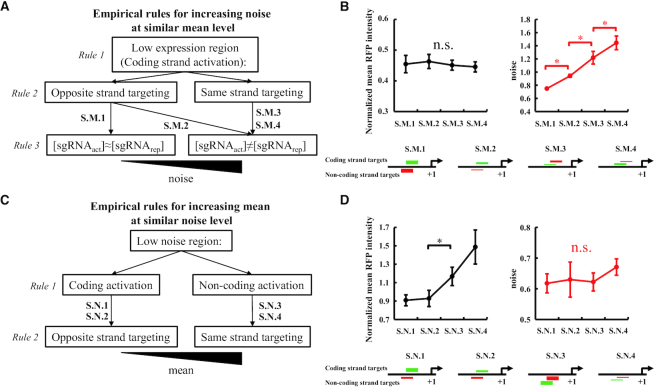
Independent tuning of expression noise and mean. (**A**) The application of the empirical rules to achieve increasing noise at a similar mean level. At each layer of the decision tree, the groups on the left are likely to have less noise than the ones on the right. (**B**) We identify a series of CRISPRar constructs that exhibit the same mean (black line) but different noise (red line. Noise level: S.M.1 < S.M.2 < S.M.3 < S.M.4. See Supplementary Table S3 for the combinations of sgRNAs). (**C**) The application of the empirical rules to achieve increasing mean at a similar noise level. At each layer of the decision tree, the groups on the left are likely to have lower expression mean than the ones on the right. (**D**) We identify CRISPRar constructs that exhibit the same noise (red line) but different mean (black line. Mean level: S.N.1 ≈ S.N.2 < S.N.3 ≈ S.N.4. See Supplementary Table S3 for the combinations of sgRNAs). For (**B**) and (**D**), the schematics of the constructs are shown below the graphs: green short lines represent activating sgRNAs, red short lines represent repressing sgRNAs. The relative concentration of the sgRNAs is represented by the thickness of the short lines. The error bars are the SEM from *n* = 6. An asterisk represents significant difference, *P* < 0.05. The n.s. represents no significant difference from ANOVA test (*P* > 0.8 for all cases).

The above empirical rules suggest that expression noise and mean may be tuned independently using CRISPRar tool. We illustrate the independent tuning of expression noise and mean in two ways. First, we use CRISPRar constructs to achieve the same expression mean, but different expression noise of RFP. We identify a three-layer decision tree according to the empirical rule: Layer 1 – low expression region; Layer 2 – the opposite and same strand targeting; Layer 3. equal and unequal [sgRNA] (Figure 4A). Targeting low expression region, which is likely a result of empirical rule 1, ensures the decoupling of expression noise and mean is significant. At a low expression region, the empirical rules 2 and 3 serve to generate distinguishable expression noise. Indeed, we have identified a series of CRISPRar constructs that exhibit the same mean but different noise (noise level: S.M.1 < S.M.2 < S.M.3 < S.M.4. Figure 4B. See Supplementary Figure S2 for more examples). We then evaluate the features of the selected constructs (schematic in Figure 4B. See Supplementary Table S3 for the sgRNA pairs). The features generally agree with the decision tree (Figure 4A). We note that the decision tree is coarse-grained and does not capture the observed quantitative difference between S.M.3 and S.M.4.

Second, we use CRISPRar constructs to achieve different expression mean but the same expression noise of RFP. Because the noise is less sensitive to the change of mean at low noise region (Supplementary Figure S3), we use CRISPRar constructs that exhibit low noise (Figure 4C). We propose a three-layer decision tree: Layer 1 – low noise region; Layer 2 – coding and non-coding strand CRISPRa; Layer 3 – the opposite and same strand targeting (Figure 4C). The empirical rule 1 ensures that the expression mean is likely to be higher for CRISPRa that targets the non-coding strand than the coding strand. The application of empirical rule 2 has two purposes. First, it selects CRISPRa that targets the coding strand and CRISPRr that targets the opposite strand, to achieve low expression noise. Second, the same-strand targeting constructs likely have a higher expression mean than the opposite-strand targeting constructs when the noise levels are similar (Figure 3B). Similarly, we have identified a series of CRISPRar constructs that exhibit different expression mean with similar noise (mean level: S.N.1 ≈ S.N.2 < S.N.3 ≈ S.N.4. Figure 4D. See Supplementary Figure S2 for more examples and Supplementary Table S3 for the sgRNA pairs). The observations are consistent with the empirical rules in the decision tree (Figure 4C). In addition, we have created 48 additional CRISPRar combinations that target a different promoter (see Supplementary Table S2 for the sequences of the new promoter and sgRNAs). We have demonstrated the tuning of noise and mean of gene expression following the same empirical rules but with the new promoter (see Supplementary Figure S4–S6 for the results and Supplementary Table S4 for new sgRNA pairs).

The CRISPRar tool may be used to study the impact of noise on bacterial evolution and survival under stress. To do this, the CRISPRar tool needs to remain stable in long term experiments. We could gain some insights into the long-term stability of CRISPRar by comparing the growth rates of the constructs (Supplementary Figure S7). For the same-mean-different-noise group (S.M. group in Figure 4B), there is no significant difference in the growth rates for three but one of the constructs. Due to the lack of correlation between the growth rates and the constructs, we speculate that the difference in the growth rate is the results but not the cause of the noise modulation. However, the exact mechanism that links the noise of our systems to the growth rate requires further investigation. For the same-noise-different-mean group (S.N. group in Figure 4D), the growth rate decreases with increasing mean as expected from the metabolic burden of gene expression. In future work, to improve the stability of these constructs, they would have to be integrated into the chromosome. In our experience with prior (30) and this work, only a small amount of dCas9 and sgRNA is required to exert a regulatory effect on gene expression. Therefore, moving from a plasmid-based to a chromosome-based system should not affect the functioning of the system. But, this speculation needs to be tested experimentally. Integrating the constructs into chromosomes, however, would reduce the ease of using such CRISPR–dCas9 constructs for the modulation of gene expression in different bacterial strains.

### The tuning of gene expression noise by CRISPRar is captured by a theoretical model

**Figure 5.**
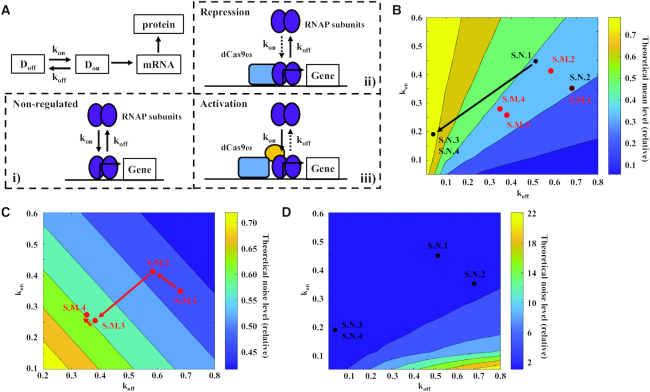
Theoretical modeling to understand the tuning of gene expression noise. (**A**) The schematic of the theoretical model. (i) In the theoretical model, the promoter of a gene can exhibit either an ON- or OFF-state corresponding to the assembly (k_on_) or disassembly (k_off_) of RNAP at the promoter. (ii) The CRISPRr can block the assembly of RNAP and therefore reduce k_on_. (iii) The CRISPRa can stabilize the RNAP and reduce k_off_. (**B**) The analytical result of expression mean is encoded into the colors of the filled contour map with respect to k_on_ and k_off_. The k_on_ and k_off_ of S.M.1–4 and S.N.1–4 (Figure 4) are estimated (see Method Section M5) and plotted onto the contour map. The black arrow indicates the increase of the mean. For (**C**) and (**D**), the color in the filled contour maps represents the analytical results of expression noise when the expression mean is the same (**C**) or different (**D**). The estimated k_on_ and k_off_ of CRISPRar constructs are plotted onto the contour maps. The red arrows indicate the increases in the noise.

To understand why CRISPRar tunes gene expression noise and mean independently, we formulate a phenomenological model. Specifically, we assume that the promoter of a gene can exhibit either an ON- or OFF-state (*D*_on_ or *D*_off_ in Figure 5A), corresponding to the assembly and disassembly of RNAP at the promoter. The switching between the ON and OFF states are described by reaction rate constants k_on_ and k_off,_ respectively (Figure 5A, panel i). Since the CRISPRr occurs by the steric hindrance of dCas9, we assume that the CRISPRr module reduces only k_on_ (Figure 5A, panel ii). In contrast, we assume that the CRISPRa decreases only k_off_ (Figure 5A, panel iii) because the ω subunit stabilizes the subunits of RNAP (24). These assumptions serve as the initial approximation of the system that we will re-examine later using the simulation results.

This approximation of the CRISPRar constructs using the state switching model allows us to use existing gene expression models in the literature (23). Based on the models, we obtain the analytical solutions that calculate relative mean and noise from approximated k_on_ and k_off_ values. To do this, we first approximate k_on_ and k_off_ of the CRISPRar constructs using the results from the characterization of CRISPRa and CRISPRr modules (Figure 1E and 2B. See Method Section M5 for details). We assume that CRISPRa and CRISPRr modules may interact in two ways. First, when two modules target the same DNA strands, they compete for the same binding sites. Second, when two modules target opposite DNA strands, one module reduces the activity of another module (See Method Section M5 for details).

Next, we use the analytical result of the expression mean (proportional to $\frac{{{{{\rm k}}_{{{\rm on}}}}}}{{{{{\rm k}}_{{{\rm on}}}} + {{{\rm k}}_{{{\rm off}}}}}}$) to investigate how relative mean changes with respect to k_on_ and k_off_ (Figure 5B. See Method Section M5 for details). We create a filled contour map where the color encodes the relative expression mean on the plane of k_on_ and k_off_ (Figure 5B). There are several regions in the contour map that exhibit a similar mean level (same color in the contour map in Figure 5B) when the values of k_on_/k_off_ are close. To compare the analytical and experimental results, we project the estimated k_on_ and k_off_ from the experimental groups that exhibit either ‘the same mean, different noise’ (S.M. groups in Figure 4B) or ‘different mean, the same noise’ (S.N. groups in Figure 4D). We find that ‘the same mean, different noise’ group does not show wide-spreading in the mean level (red dots in Figure 5B). In contrast, ‘different mean, the same noise’ group shows the change in the mean level (mean level: S.N.3 & S.N.4 > S.N.1 & S.N.2. Black dots in Figure 5B). Despite the simplicity of the model, it captures the general qualitative dynamics of the CRISPRar constructs.

We also use analytical solutions to study how the noise changes with respect to k_on_ and k_off_. For the S.M. group (the same mean, different noise), noise is proportional to $\frac{1}{{{{\bf \tau }} \cdot ( {{{{\rm k}}_{{{\rm on}}}} + {{{\rm k}}_{{{\rm off}}}}} ) + 1}}$ (theoretical noise level in Figure 5C. See Method Section M5 for details), where τ is the degradation rate constant of the reporter protein (assume τ = 1 for simplicity). We find that ‘the same mean, different noise’ group exhibits the change of gene expression noise (noise level: S.M.4 > S.M.3 > S.M.2 > S.M.1. Red dots in Figure 5C). For the S.N. group (different mean, the same noise), noise is equal to $\frac{1}{{{{\rm mean}}\ {{\rm protein}}\ {{\rm expression}}}} + \frac{{{{{\rm k}}_{{{\rm off}}}}}}{{{{{\rm k}}_{{{\rm on}}}}}} \cdot \frac{1}{{{{\bf \tau }} \cdot ( {{{{\rm k}}_{{{\rm on}}}} + {{{\rm k}}_{{{\rm off}}}}} ) + 1}}$. We find that ‘different mean, the same noise’ group does not show drastic spreading in theoretical noise level (black dots in Figure 5D). The findings again suggest that the simple model captures the qualitative dynamics of the CRISPRar constructs. However, we note that the S.M.1 and S.M.4 (Figure 5C) show some quantitative differences between the theoretical model and experimental results (Figure 4B). We will discuss the discrepancy between the modeling and experimental results in the Discussion section.

## DISCUSSION

We have developed an orthogonal tool based on CRISPR-dCas9 to tune gene expression noise in *E. coli*. The tool consists of a functionalized dCas9 (i.e. dCas9ω) and two sgRNA variants. The dCas9ω can serve as either an activator or a repressor depending on the target positions of the sgRNAs (Figure 1). This property of the dCas9ω allows us to create a library of sgRNAs that exhibit different strengths of CRISPR activation (CRISPRa) and CRISPR repression (CRISPRr). To implement the tool, one CRISPRa and one CRISPRr module are combined in a single bacterium. We create one set of 48 CRISPRar combinations for each of two different promoters (Figure 2 and Supplementary Figure S4–S6). We extract three empirical rules to guide the tuning of expression noise using the CRISPRar tool (Figure 3). Our results suggest that the expression noise and mean can be tuned independently using the CRISPRar tool. To illustrate the tuning of expression noise, we identify two sets of CRISPRar constructs: one set exhibits the same expression mean, but different expression noise; another set exhibits the same expression noise, but different expression mean (Figure 4). We have identified two decision trees based on the empirical rules (Figure 4) and a theoretical model (Figure 5) to explain the tuning of gene expression noise using the CRISPRar tool.

The extraction and demonstration of the empirical rules provide some insights into the CRISPRar tool. First, we find that the decoupling of expression noise and mean is more profound at a low expression region than at a high expression region. It is likely because the noise is intrinsically low when the expression level is strong, diminishing the detection of expression noise over measurement noise. Second, we find that the CRISPRar constructs targeting the same DNA strand generally cause higher gene expression noise than the other constructs. This observation is consistent with our speculation that the competition of CRISPRa and CRISPRr at the same promoter can increase the expression noise.

However, we do note some limitations in the empirical rules and theoretical model. First, the empirical rules are coarse-grained (Figure 3). They capture several qualitative trends (Figure 4). But, the quantitative and precise dynamics of the CRISPRar constructs require a fine-scale model supported by measurements of molecular binding to promoters. Second, we have assumed that the CRISPRr and CRISPRa only affect k_on_ and k_off_ respectively in the simplified model, and that the overlapped target sequence is critical for achieving the competition between CRISPRr and CRISPRa. The simplifying assumption is necessary due to the lack of measurements on how the assembly and disassembly of RNAP would be influenced when both CRISPRr and CRISPRa target the same promoter in the same bacterium. The competition between overlapped sgRNA has been studied for Cas9-based gene editing. Jang *et al.* have hypothesized that when two or more sgRNAs target the same DNA strand, they are likely to compete for the binding site (31). They have shown that overlapping sgRNAs causes a higher knock-in (KI) efficiency than single gRNAs. They state that the exact mechanism underlying the observation is unclear. To understand the competition mechanism of two CRISPR–Cas complexes at a promoter, the measurements may require single-molecule resolution to reveal the molecular-level interactions between CRISPRr, CRISPRa and RNAP in a single bacterium. When such data becomes available, we may be able to simulate the detailed kinetics of the CRISPR–Cas competition using stochastic chemical equations. Our simplification of the theoretical model likely underlies the quantitative discrepancy between the modeling and experimental results for S.M.1 and S.M.4 (Figures 4B and 5C).

The basic concept of CRISPRar may be applied to other organisms. In this study, we use the CRISPRar tool that consists of one functionalized dCas9 protein with two different sgRNAs to tune expression noise in *E. coli*. To achieve both activation and repression using the identical dCas9ω protein, we have to control the binding site of two sgRNAs. To implement both CRISPRa and CRISPRr in other organisms, such as mammalian cells, one may use single sgRNA companied with two dCas9 proteins that are functionalized by either a transcriptional activator (32) or repressor (33). The potential use of one sgRNA in mammalian cells may simplify the implementation of the CRISPRar tool by avoiding the need for finding different sgRNA positions on the target promoter. The CRISPRar tool may be useful in recent efforts that control gene expression noise for the study of mammalian drug resistance (34). Our CRISPRar tool represents the first orthogonal tool that can tune gene expression noise without altering the endogenous genetic components.

## Supplementary Material

gkaa451_Supplemental_File
