# Orthogonal tuning of gene expression noise using CRISPR–Cas

**DOI:** 10.1093/nar/gkaa537

**Published:** 2020-06-18

**Authors:** Fan Wu, Jiyoung Shim, Ting Gong, Cheemeng Tan

**Affiliations:** Department of Biomedical Engineering, University of California Davis, Davis, CA 95616, USA; Department of Biomedical Engineering, University of California Davis, Davis, CA 95616, USA; Department of Biomedical Engineering, University of California Davis, Davis, CA 95616, USA; Department of Biomedical Engineering, University of California Davis, Davis, CA 95616, USA

*Nucleic Acids Research*, 2020, https://doi.org/10.1093/nar/gkaa451

The first version of this article inadvertently neglected to include Ting Gong as third author. This has now been corrected online. The authors regret the error.

