# Supplementary material for: Orthogonal tuning of gene expression noise using CRISPR–Cas
Source: Nucleic Acids Res. 2020 Jun 1;48(13):e76. doi: 10.1093/nar/gkaa451 (PMC7367181; doi:10.1093/nar/gkaa451)
Supplement: gkaa451_Supplemental_File [file gkaa451_supplemental_file.pdf]

Supplementary Materials

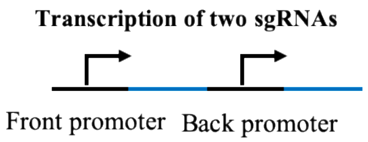

	Front pMed	Front pStr	Front pWeak
**Back pMed**	Com1	Com2	Com3
**Back pStr**		Com4	Com5
**Back pWeak**			Com6

**Com1 groups**	
Com1_A1_R2	Com1_R2_A1
Com1_A2_R2	Com1_R3_A1
Com1_A1_R3	Com1_R2_A2
Com1_A2_R3	Com1_R3_A2

**Com2 groups**	
Com2_A1_R2	Com2_R2_A1
Com2_A2_R2	Com2_R3_A1
Com2_A1_R3	Com2_R2_A2
Com2_A2_R3	Com2_R3_A2

**Com3 groups**	
Com3_A1_R2	Com3_R2_A1
Com3_A2_R2	Com3_R3_A1
Com3_A1_R3	Com3_R2_A2
Com3_A2_R3	Com3_R3_A2

**Com4 groups**	
Com4_A1_R2	Com4_R2_A1
Com4_A2_R2	Com4_R3_A1
Com4_A1_R3	Com4_R2_A2
Com4_A2_R3	Com4_R3_A2

**Com5 groups**	
Com5_A1_R2	Com5_R2_A1
Com5_A2_R2	Com5_R3_A1
Com5_A1_R3	Com5_R2_A2
Com5_A2_R3	Com5_R3_A2

**Com6 groups**	
Com6_A1_R2	Com6_R2_A1
Com6_A2_R2	Com6_R3_A1
Com6_A1_R3	Com6_R2_A2
Com6_A2_R3	Com6_R3_A2

Supplementary Figure 1. For CRISPRar constructs, two sgRNAs are transcribed from the same vector in the indicated order (front→back). Combinations of any two of the constitutive promoters that control the sgRNA are listed (Com1→Com6). Each combination is cloned to control two different sgRNAs. The first sgRNA is transcribed by the front promoter, and the second sgRNA is transcribed by the back promoter. For instance, Com2_A1_R2 indicates that sgRNA A1 is transcribed by pStr, and sgRNA R2 is transcribed by pMed. The sequence of sgRNA A1-6 and R1-3 is included in Supplementary Table 1.

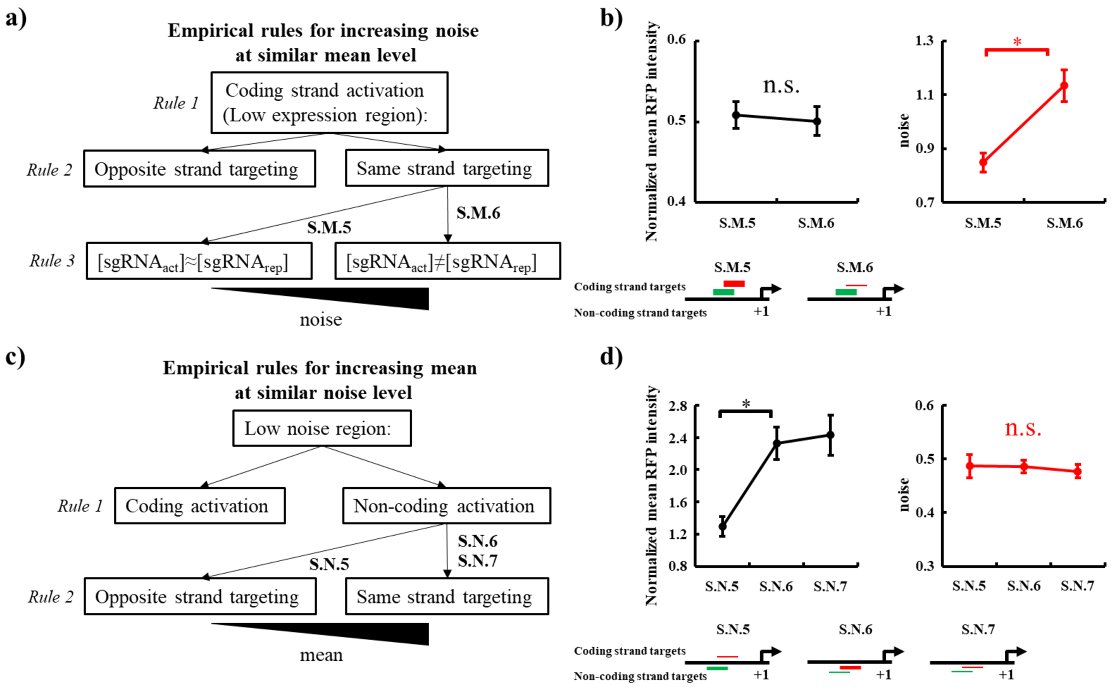

Supplementary Figure 2. Additional examples of CRISPRar that tune expression noise and mean.

a) Similar to Figure 4a, we apply a three-layer decision tree to achieve increasing noise at a similar mean level. The groups on the left of each decision-tree layer are likely to have less noise than the ones on the right.

b) We identify a group of CRISPRar constructs that exhibit the same mean (black line) but different noise (red line. Noise level: S.M.5<S.M.6). See Supplementary Table 3 for the combinations of sgRNAs.

c) Similar to Figure 4c, we apply another decision tree to achieve increasing mean at a similar noise level. At each layer of the decision tree, the groups on the left are likely to have lower expression mean than the ones on the right.

d) We identify CRISPRar constructs that exhibit the same noise (red line) but different mean (black line. Mean level: S.N.5<S.N.6≈S.N.7). See Supplementary Table 3 for the combinations of sgRNAs.

For b) and d), The schematics of the constructs are shown below the graphs: green short lines represent activation sgRNA, red short lines represent repression sgRNA. The relative concentration of the sgRNAs is represented by the thickness of the short lines. The error bars are the SEM from $n=6$. An asterisk represents significant difference from a one-tailed t-test, $p<0.05$. The n.s. represents no significant difference from ANOVA test ($p>0.8$ for Panel d).

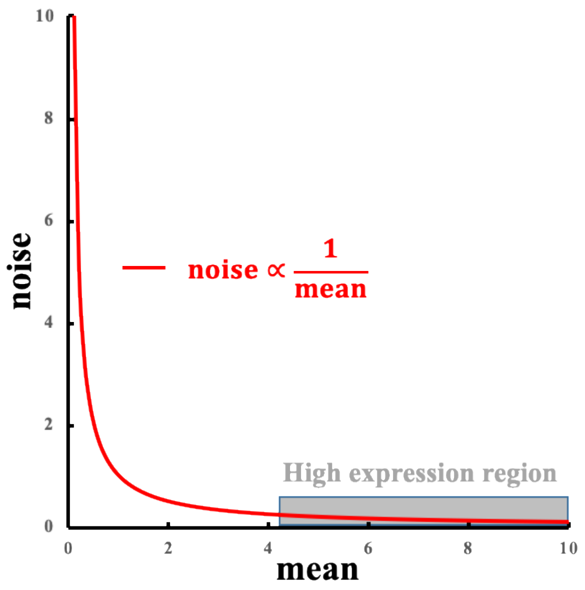

Supplementary Figure 3. The plot (red curve) shows the noise is inversely proportional to the mean in a theoretical model. At a high mean level (grey area), the change of noise is less sensitive to the change of mean.

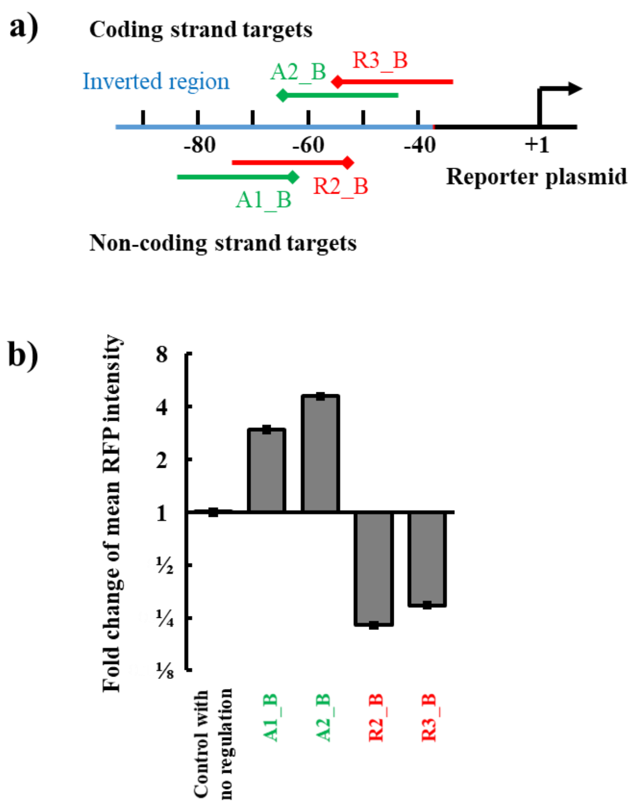

Supplementary Figure 4. Gene activation and repression using the functionalized dCas9ω protein with another promoter.

a) We create a new promoter with a different targeting region (blue color) for sgRNAs (See Supplementary Table 2 for sequence). We next construct four sgRNA variants (A1_B, A2_B, R2_B, and R3_B) for the new promoter. The new sgRNAs have similar targeting positions as the selected sgRNAs used in the main figures (See Supplementary Table 2 for the sequence of the sgRNAs). The sgRNAs guide dCas9ω to either the coding strand or the non-coding strand of the promoter. Diamond shapes represent the PAM positions of the sgRNA variants.

b) We find that A1_B & A2_B activate, and R2_B & R3_B repress the expression of the reporter. The A1_B, A2_B, R2_B, and R3_B are used to create combinatorial

CRISPRar constructs in Supplementary Figures 5 and 6. Each error bar represents the standard error of the mean (SEM) with n=6.

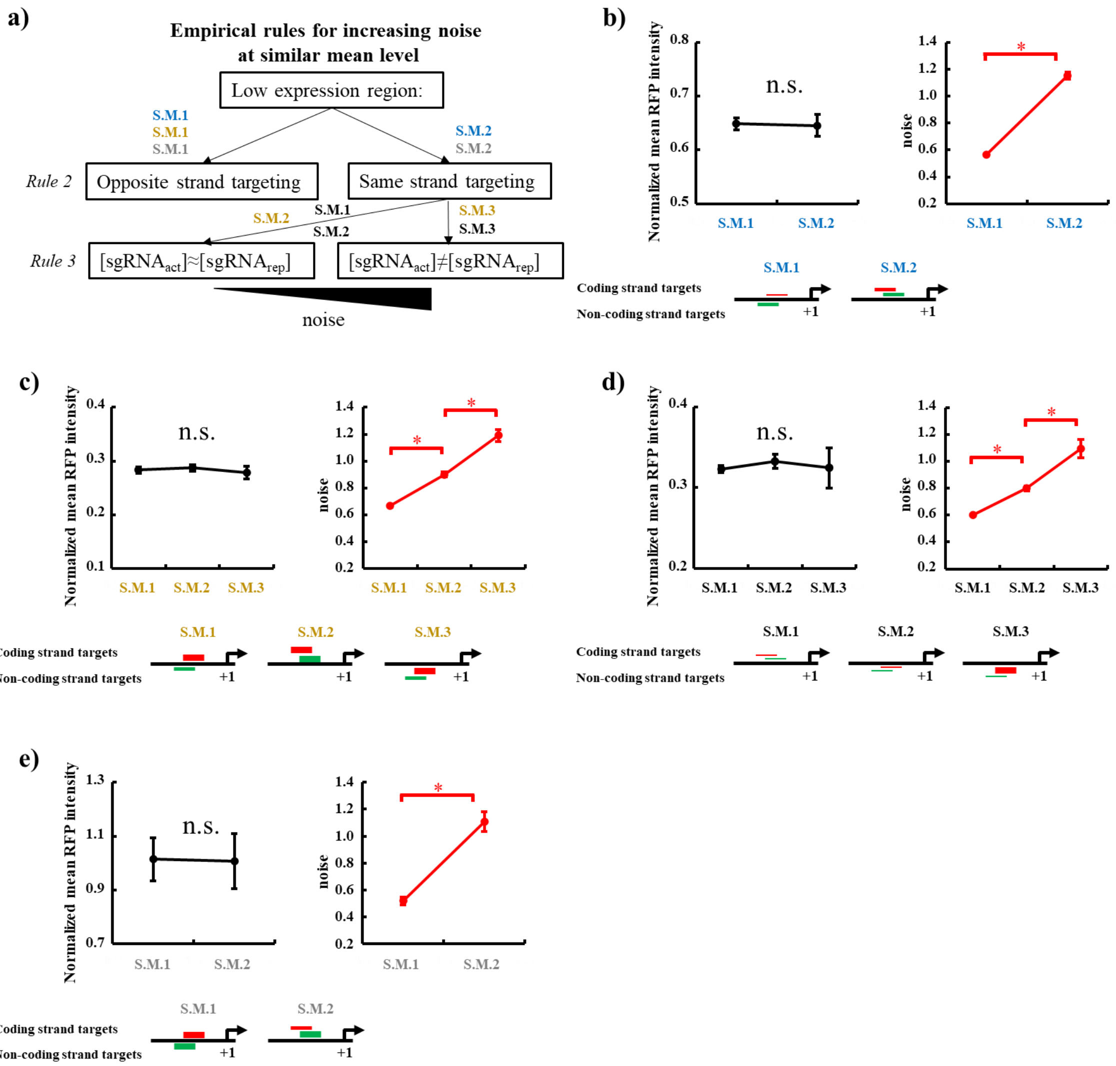

Supplementary Figure 5. To further assess the generality of the empirical rules, we construct 48 CRISPRar constructs using the new promoter and selected sgRNAs (See Supplementary Figure 4 and Supplementary Table 2) to identify "the same mean but different noise" groups.

a) We apply the same decision tree as Figure 4a. We identify four groups of constructs (**b-e**) that exhibit the same mean (black lines) but different noise (red lines. Noise level: S.M.1<S.M.2 for **b** and **e**, S.M.1<S.M.2< S.M.3 for **c** and **d)**. See Supplementary Table 4 for the combinations of sgRNAs. The coarse-grained decision

tree generally applies to the constructs we have identified here. Specifically, the constructs on the left of each layer of the decision tree are likely to have less noise than the ones on the right. The results suggest that the empirical rules are not affected by the alteration of the promoter sequence. The schematics of the constructs are shown below the graphs: green short lines represent activation sgRNA, red short lines represent repression sgRNA. The relative concentration of the sgRNAs is represented by the thickness of the short lines. The error bars are the SEM from n=6. An asterisk represents significant difference from a one-tailed t-test, $p<0.05$. The n.s. represents no significant difference from ANOVA test ($p>0.8$ for **c** and **d**).

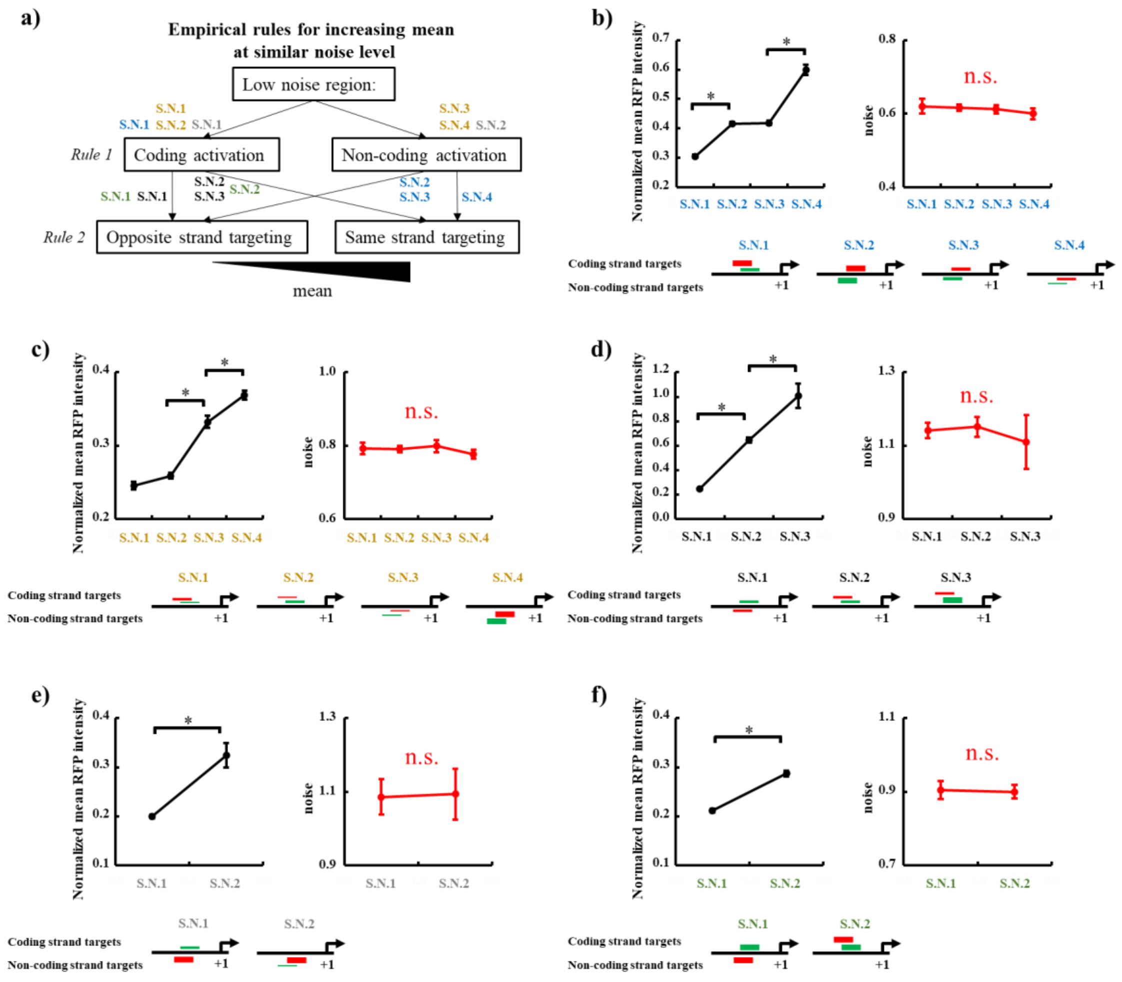

Supplementary Figure 6. Identification of "the same noise but different mean" groups from 48 CRISPRar constructs with the new promoter and selected sgRNAs (See Supplementary Figure 4 and Supplementary Table 2).

a) We apply the same decision tree as Figure 4c. We identify five groups of constructs (**b-f**) that exhibit the same noise (red lines) but different mean (black lines. Mean level: S.N.1<S.N.2≈S.N.3<S.N.4 for **b**, S.N.1≈S.N.2<S.N.3<S.N.4 for **c,** S.N.1<S.N.2<S.N.3 for **d**, S.N.1<S.N.2 for **e** and **f**. See Supplementary Table 4 for the combinations of sgRNAs).

The identified S.N. groups agree with the coarse-grained decision tree. Specifically, the constructs on the left of each layer of the decision tree are likely to have less mean than the ones on the right. The results suggest that the empirical rules are not affected by the alteration of the promoter sequence. The schematics of the constructs are shown below the graphs: green short lines represent activation sgRNA, red short lines represent repression sgRNA. The relative concentration of the sgRNAs is represented by the thickness of the short lines. The error bars are the SEM from n=6. An asterisk represents significant difference from the one-tailed t-test, $p<0.05$. The n.s. represents no significant difference from ANOVA test ($p>0.7$ for **b**-**d**).

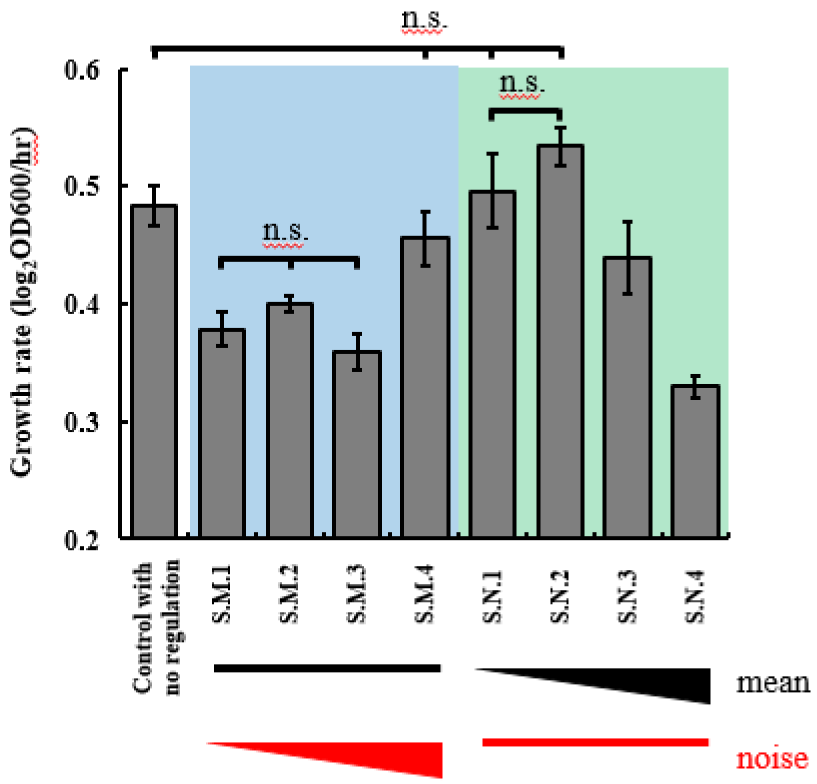

Supplementary Figure 7. The growth rate of selected S.M. and S.N. groups in Figure 4b and 4d. We measure the OD600 of selected groups in a plate reader for 14 hours. We calculate the exponential growth rate of each group (OD600 between ~0.125 and ~0.25 in our plate reader). Many constructs exhibit a growth burden (S.M.1, S.M.2, S.M.3, S.N.3 and S.N.4). We find that the growth burden is stronger as the mean intensity becomes higher for the S.N. groups (green region). The burden at a high mean level is likely due to the high expression level of the reporter protein. In contrast, the growth burden is less dependent on the changing of noise level at the same mean intensity (except S.M.4. Blue region). The error bars are the SEM from n=6. The n.s. represents no significant difference from the pair-wised one-tailed t-test, $p<0.05$.

Name	Sequence (5'->3')	PAM site distance from the transcriptional start site (bp)
R1	TGGGTTCCACCGGATACCTC	40
R2	AGGCGTCCTTTGGGTTCCAC	50
R3	AGGTATCCGGTGGAACCCAA	61
A1	CGGTTACCAAAGGCGTCCTT	60
A2	TGGAACCCAAAGGACGCCTT	71
A3	AGGACGCCTTTGGTAACCGC	81
A4	AGGTATCCTGCGGTGTCCTG	80
A5	GGGCGACCTCAGGTATCCTG	90
A6	GGGCCACCACGGGCGACCTC	100
Target sequence	gcctacggtatccaccggagacctatggcagcctccggccgccataggacacctttggttgccaagggtgacctatggtgaccatgggccaccacgggcgacctcaggtatcctgcggtgtcctgcggttaccaaaggcgtcctttgggttccaccggatacctccggac	

Supplementary Table 1. The DNA sequence of selected sgRNAs and their target region on the reporter plasmid (Figure 1-5 and Supplementary Figure 2).

Name	Sequence (5'->3')	PAM site distance from the transcriptional start site (bp)
R2_B	CGGAGGCTGCCATAGGTCTC	52
R3_B	CGGTATCCACCGGAGACCTA	60
A1_B	AGGTGTCCTATGGCGGCCGG	69
A2_B	CGGAGACCTATGGCAGCCTC	70
Target sequence	gtccggaggtatccggtggaacccaaaggacgcctttggtaaccgcaggacaccgcaggatacctgaggtcgcccgtggtggcccatggtcaccataggtcacccttggcaaccaaaggtgtcctatggcggccggaggctgccataggtctccggtggataccgtaggc	

Supplementary Table 2. The DNA sequence of the altered target region of the new promoter and selected sgRNAs (Supplementary Figure 4-6).

Name	Activation sgRNA index	Repression sgRNA index	Name	Activation sgRNA index	Repression sgRNA index
S.M.1 in Figure 4b	A1	R3	S.N.1 in Figure 4d	A1	R3
S.M.2 in Figure 4b	A1	R3	S.N.2 in Figure 4d	A1	R3
S.M.3 in Figure 4b	A2	R3	S.N.3 in Figure 4d	A1	R2
S.M.4 in Figure 4b	A2	R3	S.N.4 in Figure 4d	A1	R2
S.M.5 in Supplementary Figure 2	A2	R3	S.N.5 in Supplementary Figure 2	A1	R3
S.M.6 in Supplementary Figure 2	A2	R3	S.N.6 in Supplementary Figure 2	A1	R2
			S.N.7 in Supplementary Figure 2	A1	R2

Supplementary Table 3. The index of sgRNAs used in selected groups from Figure 4 and Supplementary Figure 2.

Name	Activation sgRNA index	Repression sgRNA index	Name	Activation sgRNA index	Repression sgRNA index	Name	Activation sgRNA index	Repression sgRNA index
Supp. Fig. 5b			*Supp. Fig. 6b*			*Supp. Fig. 6e*		
S.M.1	A1_B	R3_B	S.N.1	A2_B	R3_B	S.N.1	A2_B	R2_B
S.M.2	A2_B	R3_B	S.N.2	A1_B	R3_B	S.N.2	A1_B	R2_B
Supp. Fig. 5c			S.N.3	A1_B	R3_B	*Supp. Fig. 6f*		
S.M.1	A1_B	R3_B	S.N.4	A1_B	R2_B	S.N.1	A2_B	R2_B
S.M.2	A2_B	R3_B	*Supp. Fig. 6c*			S.N.2	A2_B	R3_B
S.M.3	A1_B	R2_B	S.N.1	A2_B	R3_B			
Supp. Fig. 5d			S.N.2	A2_B	R3_B			
S.M.1	A2_B	R3_B	S.N.3	A1_B	R2_B			
S.M.2	A1_B	R2_B	S.N.4	A1_B	R2_B			
S.M.3	A1_B	R2_B	*Supp. Fig. 6d*					
Supp. Fig. 5e			S.N.1	A2_B	R2_B			
S.M.1	A1_B	R3_B	S.N.2	A2_B	R3_B			
S.M.2	A2_B	R3_B	S.N.3	A2_B	R3_B			

Supplementary Table 4. The index of sgRNAs used in selected groups from Supplementary Figure 5 and 6.

Parameter	Definition of the parameter	Example of equation involved	Description of the equation involved
**k_a and k_r**	Maximum inhibition rate	$k_{off} = k_{off,wt}(1 - \frac{k_a[A]}{K_a + [A]} \cdot int_2)$ $k_{on} = k_{on,wt}(1 - \frac{k_r[R]}{K_r + [R]} \cdot int_1)$	Empirical equations that describe the inhibition of k_{off} or k_{on} by CRISPR activation or repression
**K_a and K_r**	Apparent value of half-maximum constant due to competitive binding	Same as above	Same as above
**K_{ao} and K_{ro}**	Half-maximum constant	$K_a = K_{ao}(1 + \frac{[R]}{K_i})$ $K_r = K_{ro}(1 + \frac{[A]}{K_i})$	Equation for apparent half-maximum constant due to competitive binding
**K_i**	Dissociation constant of binding competitor	Same as above	Same as above
**Int_1 and Int_2**	Modulator for opposite-strand interaction.	$int_1 = \frac{1}{1 + K_{int1}[A]}$ $int_2 = \frac{1}{1 + K_{int2}[R]}$	Empirical equations that describe the interaction between CRISPR activation and CRISPR repression when targeting opposite-strands of DNA. The values of int_1 and int_2 are less or equal to 1.
**K_{int1} and K_{int2}**	Empirical constants in int_1 and int_2	Same as above	Same as above

Supplementary Table 5. The definition of parameters used in the theoretical model.
